# A Hybrid Detection Technique for Electro-Mechanical Scanning Radars Integrating Omnidirectional Surveillance and Sector-Specific Energy Focusing

**DOI:** 10.3390/s26217008

**Published:** 2026-11-05

**Authors:** Dong Ni, Yu Fang, Peng Zhang, Xinguo Zhu

**Affiliations:** Nanjing Research Institute of Electronics Technology, Nanjing 210039, China

**Keywords:** electro-mechanical scanning radar, energy focusing, specific sector, omnidirectional surveillance

## Abstract

Air surveillance radars typically operate in two distinct modes—omnidirectional surveillance and sector-specific surveillance—which are inherently incompatible in conventional designs. To overcome this limitation, this paper proposes a novel flexible detection technique for electro-mechanical scanning radars that integrates omnidirectional surveillance with sector-specific energy focusing. The operational constraints and capacity boundaries of the proposed hybrid mode are rigorously analyzed and quantified through a mathematical framework. Furthermore, UAV-based calibration flight tests are conducted to validate the feasibility of the proposed technique. Experimental results demonstrate that the hybrid mode achieves a significant detection range extension in the energy-focused sector while maintaining continuous omnidirectional coverage in other sectors. This approach therefore offers a practical and effective novel operational mode, which can significantly enhance the battlefield adaptability and detection performance of electro-mechanical scanning radars.

## 1. Introduction

A radar is an electromagnetic system for detection, location, and sometimes for recognition of target objects, which operates by transmitting electromagnetic signals, receiving echoes from target objects within its volume of coverage, and extracting location and other information from the echo signals [1].

Currently, electro-mechanical scanning radars that combine both phase-scanning and mechanical scanning have become the mainstream radar architecture. In recent years, research into the theories and applications of such radars has attracted increasing attention [2,3,4,5,6,7,8]. For instance, a design concept was proposed and studied to satisfy the requirements for weather observation using a multifunction polarimetric phased-array radar, capable of achieving a volume update time of approximately one minute while meeting current aviation standards [2]. A sensor selection scheme for multi-target tracking (MTT) in a phased-array radar (PAR) network operating in hostile environments was developed in [4]. The proposed scheme adaptively adjusts the limited number of beams in each PAR to improve MTT performance while reducing the tracking capability available to enemy targets. In [5], initial weather measurements using a cylindrical polarimetric phased array radar demonstrator developed at the University of Oklahoma are presented. The measurements are first qualitatively validated, followed by a quantitative comparison of two scan modes, with derived error statistics discussed in detail. Reference [6] provides an overview of the interrelated aspects of the digital beamforming trade space, including data distribution within the array, data processing, calibration, and the comparison between fixed and adaptive beamforming.

Recent advances in phased-array radar resource management have demonstrated the potential of intelligent scheduling algorithms to adaptively allocate dwell time and beam resources under dynamic operational scenarios [9,10].

The integration of mechanical rotation and electronic phase scanning has been increasingly studied, leading to hybrid scanning strategies that balance wide-area coverage and directional energy concentration [11,12].

Dwell time optimization and coherent integration techniques have been shown to significantly improve radar detection range in specific sectors by concentrating transmitted energy [13,14].

The patent CN111813511A proposed a resource scheduling method for rotating phased-array radars that enables azimuth-sector scheduling, electronic beam steering during mechanical rotation, and retrace-type task insertion.

For electro-mechanical scanning radars, particularly in the field of air surveillance, two typical operation modes are commonly employed. One is the omnidirectional surveillance mode, in which the radar mechanically rotates in azimuth to detect targets within a 360-degree coverage area, generally with an elevation coverage not exceeding 30°. The other is the sector-specific surveillance mode, also referred to as the energy-focus mode, in which the radar stops rotating and points in a fixed direction, detecting targets within a limited azimuth sector via phase scanning (typical azimuthal scan ranges are ±30°, ±45°, or ±60°).

The omnidirectional surveillance mode enables the detection of airborne targets across a full 360° azimuth range, providing uniform detection capability in all directions (ignoring obstruction by obstacles) and requiring only a relatively simple scheduling system. In contrast, the sector-specific surveillance mode is employed to detect targets within a predetermined and limited azimuth sector, offering improved search data rates and extended detection ranges. However, these two operation modes are mutually incompatible in conventional radar systems, as the radar must sacrifice either continuous 360° coverage or the ability to concentrate energy in a particular direction.

When operating in omnidirectional surveillance mode, it is often desirable for a radar to extend its detection range as far as possible within certain sectors of interest, particularly to detect stealth or jamming targets [1]. To the best of our knowledge, existing works on rotating phased-array radar resource management have not explicitly addressed the joint optimization of back-scanning and pre-scanning within a coordinated mechanical-electronic scanning framework. As a contribution to fill this gap, this paper proposes a flexible detection technique for electro-mechanical scanning radars that integrates omnidirectional surveillance with sector-specific energy focusing. Unlike existing approaches that treat these two modes as separate, the proposed method achieves both continuous azimuth coverage and enhanced detection range in a designated sector simultaneously, thereby improving the radar’s operational flexibility and tactical effectiveness.

The novelty of the present work lies in three specific aspects that are not addressed in CN111813511A: (i) a symmetric back-scanning/pre-scanning dwell-time redistribution scheme that enables detection range extension in a designated sector while maintaining omnidirectional coverage elsewhere; (ii) a rigorous analytical derivation of the system’s capability boundaries, providing closed-form constraints for dwell-time allocation under the coordinated scanning framework; and (iii) experimental validation using UAV calibration flight tests, which demonstrates a practical range improvement of approximately 28–30% in the focused sector. In contrast, CN111813511A focuses on task scheduling and retrace-type insertion without providing a quantitative framework for dwell-time redistribution or experimental demonstration of range extension.

## 2. Technology Approach

The proposed resource coordination mechanism utilizes two complementary scanning modalities: mechanical rotation provides coarse azimuth coverage over the full 360° field of regard, while electronic phase scanning enables rapid beam steering within each angular sector. The electronic scanning capability is essential for two key functions: (i) back-scanning, i.e., revisiting a previously passed sector to extend the dwell time on a detected target; (ii) pre-scanning, i.e., briefly inspecting an upcoming sector ahead of the mechanical rotation to obtain early target detection. Both functions rely on the ability of the phased-array antenna to electronically steer the beam within a sector without moving the mechanical platform.

Consider a radar operating in the typical omnidirectional surveillance mode, with its antenna array rotating clockwise at a constant angular speed. Let the azimuthal beamwidth of the radar antenna be denoted as *θ*, the dwell time per beam position be *t*, and the detection range in each azimuthal direction be *R*.

As illustrated in Figure 1, the full 360° azimuth coverage is divided into 2N + 1 sectors. Sector 0 (the green zone, with azimuth span *θ*_2_) is a designated sector requiring a longer detection range compared to other sectors. Let *t*_2_ represent the dwell (observation) time per beam position in sector 0, and *R*_2_ denote the achievable detection range in this sector. The azimuthal span of each sector from 1 to N − 1 and from N + 1 to 2N is assumed to be *θ*_1_, while the span of sector N is *θ*_N_ (0 < *θ*_N_ ≤ *θ*_1_). Here, *θ*_1_ is generally no greater than the maximum scanning angle of the antenna *θ*_s_. The detection range for sectors 1 through 2N (i.e., outside the specific sector) is denoted as *R*_1_.

The total dwell time required for sector 0 is *t*_2_*θ*_2_/*θ*, which exceeds the dwell time *tθ*_2_/*θ* allocated per beam position within this sector under conventional mechanical scanning. The additional time (*t*_2_ − *t*)*θ*_2_/*θ* can be compensated solely by the two sectors adjacent to sector 0 (i.e., sectors 1 and 2N), whose azimuthal coverage does not exceed the maximum phase-scanning angle of the antenna, through beam back-scanning or pre-scanning. This compensation mechanism is the core of the proposed technique. A detailed explanation is as follows. When the mechanical axis of the antenna is positioned in sector 1, part of the dwell time in sector 1 is used for detection in sector 0 via beam back-scanning toward sector 0. Similarly, when the mechanical axis is positioned in sector 2N, part of the dwell time in sector 2N is used for detection in sector 0 via beam pre-scanning toward sector 0.

The increase in dwell time leads to an extended detection range in sector 0. Conversely, the reduction in dwell time causes a decreased detection range in sector 1. However, the dwell time in sector 1 can be supplemented by beam back-scanning from sector 2 to sector 1. Similarly, the dwell time in sector 2 can be supplemented by beam back-scanning from sector 3 to sector 2, and so forth, up to sector N − 1, whose dwell time can be supplemented by beam back-scanning from sector N to sector N − 1. Likewise, the dwell time in sector 2N can be supplemented by beam pre-scanning from sector 2N − 1 to sector 2N, and this pattern continues.

The back-scanning/pre-scanning mechanism provides an extension of the effective observation time for a given angular sector. This additional time is used to transmit and receive additional radar pulses within the same sector. Specifically:

- The original (conventional) dwell time in a sector is T_dwell,conv_, yielding N_conv_ = PRF × T_dwell,conv_ integrated pulses.
- Through back-scanning/pre-scanning, the effective dwell time is increased to T_dwell,enh_ = T_dwell,conv_ + ΔT, where ΔT is the extra time borrowed from adjacent sectors.
- The number of integrated pulses increases to N_enh_ = PRF × T_dwell,enh_.
- Under the coherent integration assumption, the integrated SNR improves by a factor of N_enh_/N_conv_.
- This SNR improvement translates into an extended detection range according to the R^4^ ∝ T_dwell_ relationship derived above.

According to the radar equation, when other parameters are fixed, the fourth power of the radar detection range is positively correlated with the dwell time [15]. Therefore, compared with detection range *R* in conventional mechanical scanning, the detection range in a specific sector with energy focusing is extended by a factor of

(1) $$\frac{R_{2}}{R}=\sqrt[{4}]{\frac{t_{2}}{t}}$$

The total available dwell time across sectors 1 to 2N is 2*t*π/*θ* − *t*_2_*θ*_2_/*θ*. Assuming the dwell time is evenly distributed among these sectors, the average dwell time per beam position becomes

(2) $$t_{1}=\frac{\frac{2\pi }{\theta }t-\frac{\theta_{2}}{\theta }t_{2}}{\frac{2\pi -\theta_{2}}{\theta }}=\frac{2\mathrm{\pi t}-\theta_{2}t_{2}}{2\pi -\theta_{2}}$$

Thus, compared with detection range *R* in conventional mechanical scanning, the detection range in sectors 1 to 2N without energy focusing is reduced by a factor of

(3) $$\frac{R_{1}}{R}=\sqrt[{4}]{\frac{t_{1}}{t}}$$

In summary, when an electro-mechanical scanning radar operates in the omnidirectional surveillance mode, the dwell time in a specific sector can be increased through beam back-scanning or pre-scanning, thereby achieving a greater detection range via energy focusing. Conversely, the dwell time in sectors 1 to 2N without energy focusing is correspondingly reduced, resulting in a shorter detection range in these sectors.

The notations and definitions of variables are provided in Appendix A.

## 3. Constraints

As shown in Figure 1, when the mechanical axis of the antenna is positioned within any sector of the right semicircle (sectors 1 to N), a portion of the dwell time is allocated to beam back-scanning toward the previous sector in order to focus energy on sector 0. Similarly, when the mechanical axis falls within any sector of the left semicircle (sectors N + 1 to 2N), part of the dwell time is used for beam pre-scanning to the next sector, also for the purpose of energy focusing on sector 0.

Due to symmetry, it is sufficient to consider only the time resource allocation within the right semicircle. As previously defined, the angular span of each sector from 1 to N − 1 is *θ*_1_, while the span of sector N is *θ*_N_ (0 < *θ*_N_ ≤ *θ*_1_). Where *θ*_1_ denotes the maximum scanning angle of the antenna.

Figure 2 illustrates the time resource allocation scheme for radar detection that integrates omnidirectional surveillance with sector-specific energy focusing.

To satisfy the detection range requirement in sector 0 (characterized by *θ*_2_ and *t*_2_), when the mechanical axis of the antenna falls within sector 1, the duration required for beam back-scanning to sector 0—thereby increasing the dwell time in sector 0—is given by

(4) $$T_{1}=\frac{\theta_{2}}{2\theta }(t_{2}-t)$$

Meanwhile, the effective dwell time in sector 1 is reduced, resulting in a frame time per beam position less than *t*_1_. This deficit can be compensated when the mechanical axis falls within sector 2, during which beam back-scanning from sector 2 to sector 1 occurs. The required duration for this back-scan is

(5) $$T_{2}=\frac{\theta_{1}}{\theta }t_{1}-(\frac{\theta_{1}}{\theta }t-T_{1})=T_{1}-\frac{\theta_{1}}{\theta }(t-t_{1})$$

By extension, the required duration for beam back-scanning from sector *n* to sector *n* − 1 is

(6) $$T_{n}=T_{n-1}-\frac{\theta_{1}}{\theta }(t-t_{1})$$

From the above recurrence, it follows that *T_n_* − *T_n_*_−1_ = −(*t* − *t*_1_)*θ*_1_/*θ*. Obviously, the arithmetic sequence {*T_n_*} is monotonically decreasing. Therefore, *T_n_* can be expressed as

(7) $$T_{n}=\frac{\theta_{2}}{2\theta }(t_{2}-t)-(n-1)\frac{\theta_{1}}{\theta }(t-t_{1})\;\;\;\;n=1,2,\ldots N$$

To ensure the feasibility of radar detection combining omnidirectional surveillance with sector-specific energy focusing, the following constraints must be satisfied.

Constraint 1: Half of the additional time resource required by the specific sector must not exceed the dwell time allocated to sector 1 under mechanical scanning, i.e.,

(8) $$T_{1}\leq \frac{\theta_{1}}{\theta }t$$

Substituting *T*_1_ = (*t*_2_ − *t*)*θ*_2_/(2*θ*) into (8), this constraint becomes (*t*_2_ − *t*)*θ*_2_/(2*θ*) ≤ *tθ*_1_/*θ*.

In the most extreme case, where equality holds in (8), the entire dwell time within sector 1 under mechanical scanning is used for back-scanning to sector 0. By symmetry, the entire dwell time within sector 2N under mechanical scanning is used for pre-scanning to sector 0.

Similarly, the additional time resource required by sector 1 must not exceed the dwell time within sector 2 under mechanical scanning, i.e., *T*_2_ ≤ *tθ*_1_/*θ*.

The additional time resource required by sector N − 2 must not exceed the dwell time within sector n + 1 under mechanical scanning, i.e., *T*_N−1_ ≤ *tθ*_1_/*θ*.

In particular, the additional time resource required by sector N − 1 must not exceed the dwell time within sector N under mechanical scanning, i.e., *T*_N_ ≤ *tθ*_N_/*θ*, (0 < *θ*_N_ ≤ *θ*_1_).

Since the sequence {*T_n_*} is monotonically decreasing, the condition *T_n_* ≤ *tθ*_1_/*θ* holds for all *n* = 1, 2, …, N − 1 as long as *T*_1_ ≤ *tθ*_1_/*θ* is satisfied. For the case *n* = N, the condition *T*_N_ ≤ *tθ*_N_/*θ* is ensured by Constraint 2 below.

In summary, Constraint 1 governs both the maximum achievable detection range in the energy-focused sector (determined by *T*_1_) and the search continuity in sectors 1 through 2N.

Constraint 2: The effective dwell time per beam position in sector N, after excluding the time used for back-scanning, must equal the average frame time of sectors 1 to N, i.e.,

(9) $$t-\frac{\theta }{\theta_{N}}T_{N}=t_{1}$$

Substituting *T*_N_ = (*t*_2_ − *t*)*θ*_2_/(2*θ*) − (N − 1)(*t* − *t*_1_)*θ*_1_/*θ* into (9), the constraint becomes

(10) $$t_{1}=\frac{2\mathrm{\pi t}-\theta_{2}t_{2}}{2\pi -\theta_{2}}$$

Similarly, for sectors 1 through N − 1, the effective dwell time per beam position after excluding back-scanning time is equal to the average frame time, i.e.,

(11) $$t-\frac{\theta }{\theta_{1}}T_{N-1}+\frac{\theta }{\theta_{1}}T_{N}=t-(t-t_{1})=t_{1}$$

In summary, Constraint 2 determines the achievable detection range in sectors 1 through 2N, as governed by the average dwell time *t*_1_.

## 4. Capability Boundaries

The capability boundaries of radar detection combining omnidirectional surveillance and sector-specific energy focusing can be derived from Constraint 1 and Constraint 2.

From Constraint 1, we have (*t*_2_ − *t*)*θ*_2_/(2*θ*) ≤ *tθ*_1_/*θ*, which can be rewritten as *t*_2_/*t* ≤ 1 + 2*θ*_1_/*θ*_2_. Given that, when other parameters are fixed, the fourth power of the radar detection range is positively correlated with the dwell time [15], it follows that

(12) $$\frac{R_{2}}{R}\leq \sqrt[{4}]{1+\frac{2\theta_{1}}{\theta_{2}}}$$

The physical interpretation of (12) is as follows. If the maximum phase-scanning angle of the antenna is held constant, a smaller angular coverage of the specific sector allows for a greater achievable detection range within that sector. Conversely, if the angular coverage of the specific sector is fixed, a larger maximum phase-scanning angle of the antenna also enables a greater detection range within that sector. Equation (12) is illustrated in Figure 3.

For sectors 1 through 2N, the capability boundaries can be derived from Constraint 2. According to Constraint 2, we have *t*_1_ = (*t*π/*θ* − *t*_2_*θ*_2_/(2*θ*))*θ*/(π − *θ*_2_/2), which can be rewritten as *t*_1_ /*t* = 1 − 2*θ*_1_/(2π − *θ*_2_).

Since the fourth power of the radar detection range is proportional to the dwell time when other parameters are fixed [15], we obtain

(13) $$\frac{R_{1}}{R}\geq \sqrt[{4}]{1-\frac{2\theta_{1}}{2\pi -\theta_{2}}}$$

The physical interpretation of (13) is as follows. If the maximum phase-scanning angle of the antenna is held constant, a smaller angular coverage of the specific sector allows for a greater detection range in sectors 1 through 2N. Moreover, if the angular coverage of the specific sector is fixed, a larger maximum phase-scanning angle of the antenna leads to a reduced detection range in sectors 1 through 2N. Equation (13) is plotted in Figure 4.

These capability boundaries provide important design guidelines for radar engineers. For a given set of operational requirements (e.g., desired detection range in the focused sector and acceptable range reduction in other sectors), the values of *θ*_1_ and *θ*_2_ can be selected to meet the trade-off. This quantitative framework thus facilitates the practical deployment of the proposed hybrid detection technique.

## 5. Experimental Verification

To validate the feasibility of the proposed technique, a series of calibration flight tests were designed and conducted. An octocopter unmanned aerial vehicle (UAV) with a radar cross-section (RCS) of 0.5 m^2^ served as the target. An air surveillance radar equipped with both mechanical and phase-scanning (i.e., electro-mechanical scanning) capabilities was employed to detect the UAV, and the maximum detection ranges were recorded.

Assume that the radar operates at a rotation rate of 6 rpm, with a maximum phase-scanning angle of *θ*_1_ = 45°, an azimuthal beamwidth of *θ* = 1.8°, a dwell time per beam position of *t* = 50 ms, and a detection range of *R* = 75 km in the omnidirectional surveillance mode (for targets with an RCS of 0.5 m^2^). When operating in the omnidirectional surveillance mode, a specific azimuth sector spanning from 0° to 30° (*θ*_2_ = 30°) is designated for energy focusing, leveraging the radar’s electro-mechanical scanning capability. Based on the preceding analysis, the theoretical detection range in each sector is illustrated in Figure 5a (ignoring obstructions). As shown in Figure 5a, the detection range in the azimuth sectors from 30° to 360° is reduced from 75 km to 69 km, while the detection range in the key sector from 0° to 30° is correspondingly increased from 75 km to 105 km—an improvement of approximately 40%. The main operating parameters of the radar are given in Appendix B.

In the experimental setup, the UAV was maneuvered to approach the radar along a radial trajectory. To ensure a comprehensive verification of the radar’s detection range, the UAV maintained an altitude exceeding 700 m, with a maximum slant range surpassing 110 km.

The calibration flight tests were conducted in two stages. In the first stage, the radar operated in the omnidirectional surveillance mode, performing a full 360° mechanical azimuth scan. Subsequently, the UAV approached the radar radially along a predetermined azimuth of 5°. Upon detection, the measured range was recorded as the maximum detection range for that specific direction. The “maximum detection range” is defined as the range at which the radar first establishes a stable target track. This procedure was repeated sequentially for azimuths of 25°, 95°, 115°, 185°, 205°, 275°, and 295°. The maximum detection ranges obtained for each direction are summarized in Table 1.

The RCS value of 0.5 m^2^ was obtained from anechoic chamber measurements at the nose-on aspect of the UAV. In the experiment, the UAV flew on a radial trajectory directly toward the radar. Therefore, the target aspect remained approximately nose-on throughout the measurement, and the RCS remained consistent with the 0.5 m^2^ value.

In the second stage, the radar operated in a hybrid mode combining omnidirectional surveillance with energy focusing in a specific sector. As the UAV approached the radar radially along various azimuths, the maximum detection ranges were recorded, as shown in Table 1 and plotted in Figure 5b.

A comparison between the measured results and theoretical predictions (Figure 5 and Table 1) reveals strong agreement in the omnidirectional surveillance mode. Notably, the detection ranges in the targeted sector (azimuths 5° and 25°) with energy focusing are significantly greater than those in the same sector without focusing—confirming a range extension of approximately 28–30% (from 75 km to about 96 km). Conversely, the detection ranges in the non-targeted sectors (95°, 115°, 185°, 205°, 275°, and 295°) are reduced compared to those in the omnidirectional mode. These results demonstrate the effectiveness of the proposed hybrid detection strategy. Furthermore, the measured detection ranges with energy focusing are slightly lower than the theoretical values. This discrepancy is attributed to antenna phase-scanning losses [16]. A quantitative analysis of these scan losses is beyond the scope of this paper.

It should be noted that only one flight run was performed for each investigated azimuth. Therefore, the experimental results should be regarded as a proof-of-concept validation rather than a statistical characterization of detection range. The measurements demonstrate the expected trend of increased range in the prioritized sector and reduced range in the remaining sectors, but the absolute range values and their repeatability require further experimental investigation.

## 6. Conclusions

This study presents a hybrid electro-mechanical scanning radar technique that integrates omnidirectional surveillance with sector-specific energy focusing. The working principle and operational constraints are rigorously analyzed, leading to the derivation of the system’s performance boundaries—including the maximum achievable range extension in the focused sector and the corresponding range reduction in other sectors. The experimental results confirm the practical feasibility of the proposed technique and show a consistent trend of range extension in the focused sector. Further experimental studies with repeated flight tests are needed to establish the statistical characteristics of the detection-range performance. This hybrid detection technique offers a significant improvement in operational flexibility for air surveillance radars, enabling them to simultaneously meet the demands of broad-area monitoring and prioritized long-range detection in specific directions.

## Figures and Tables

**Figure 1:**
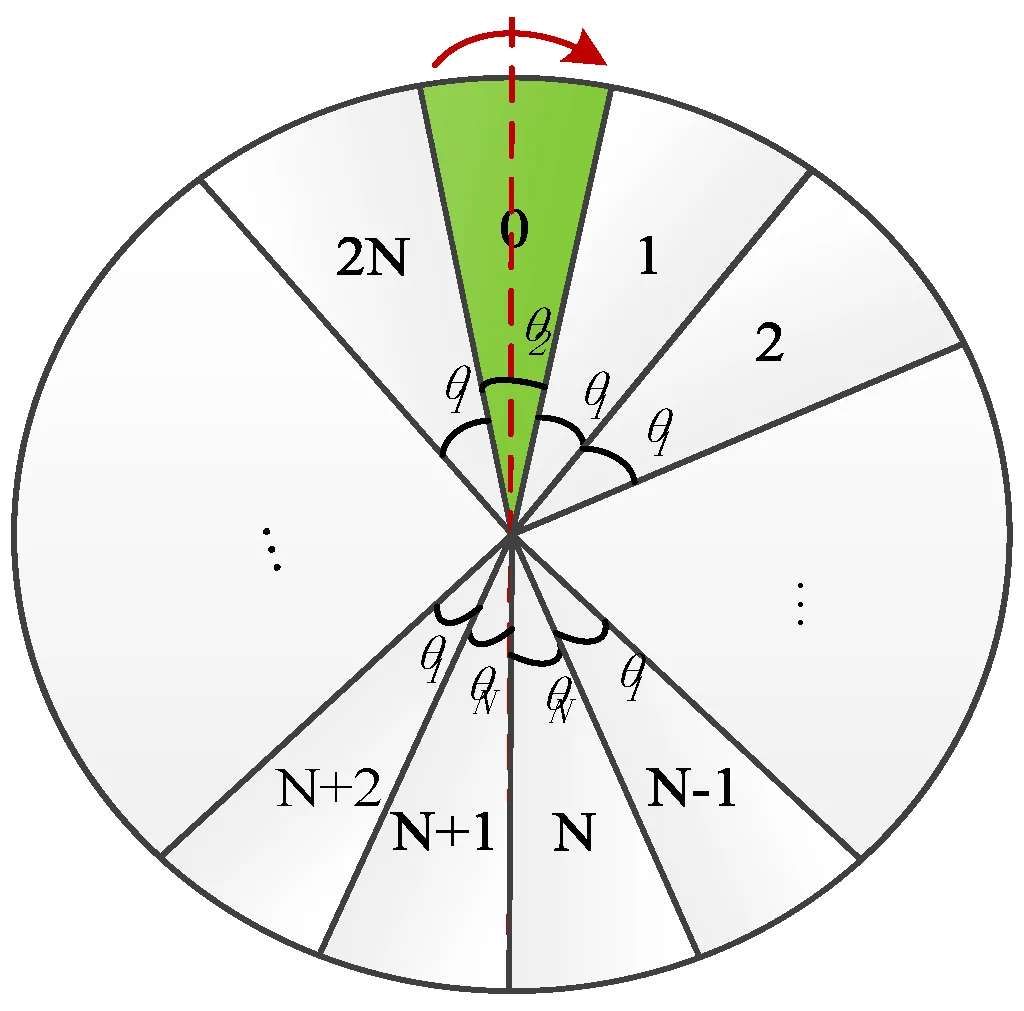
Schematic diagram of detection sectors.

**Figure 2:**
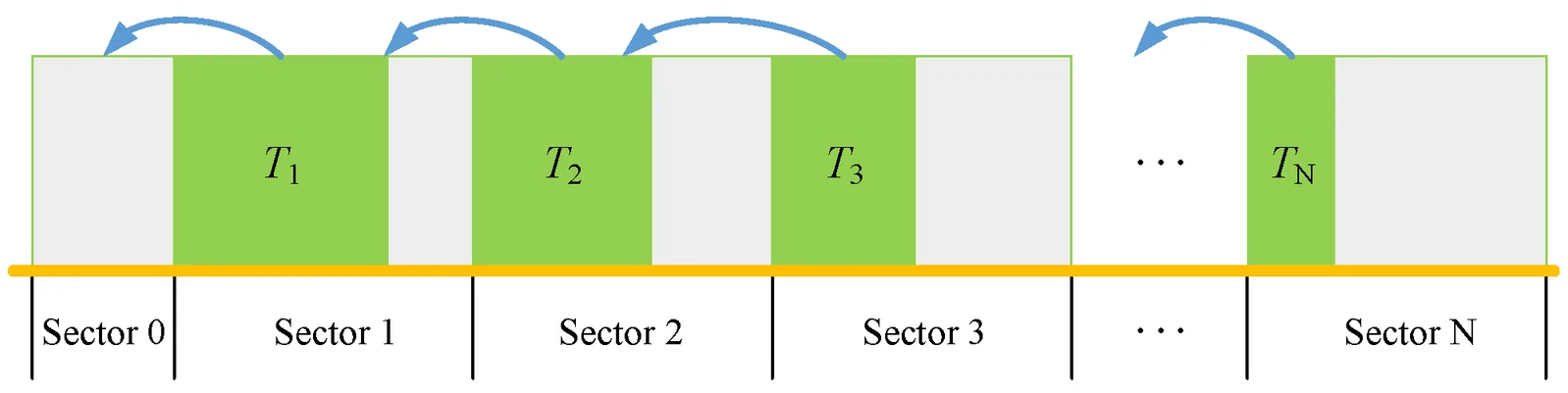
Time resource allocation for radar detection combining omnidirectional surveillance and sector-specific energy focusing.

**Figure 3:**
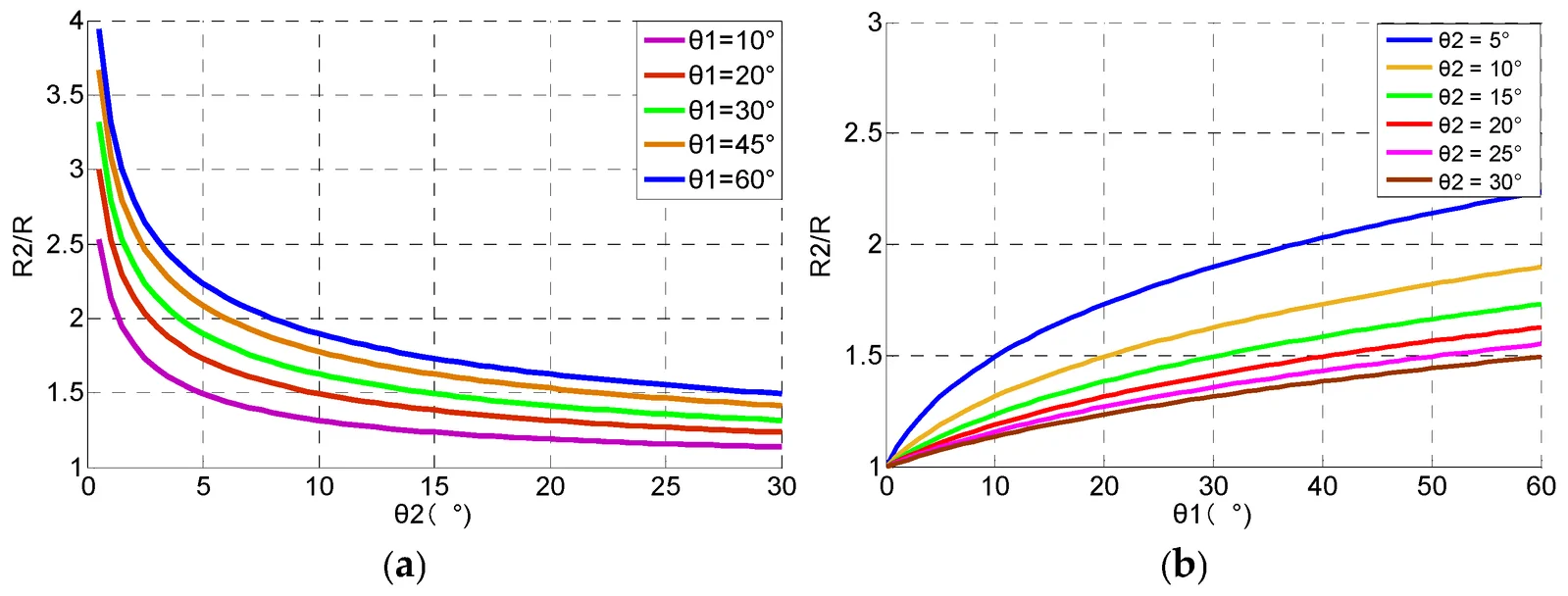
Relationship between the maximum detection range in a specific sector and *θ*_1_ and *θ*_2_. (**a**) with *θ*_2_ as the horizontal axis, (**b**) with *θ*_1_ as the horizontal axis.

**Figure 4:**
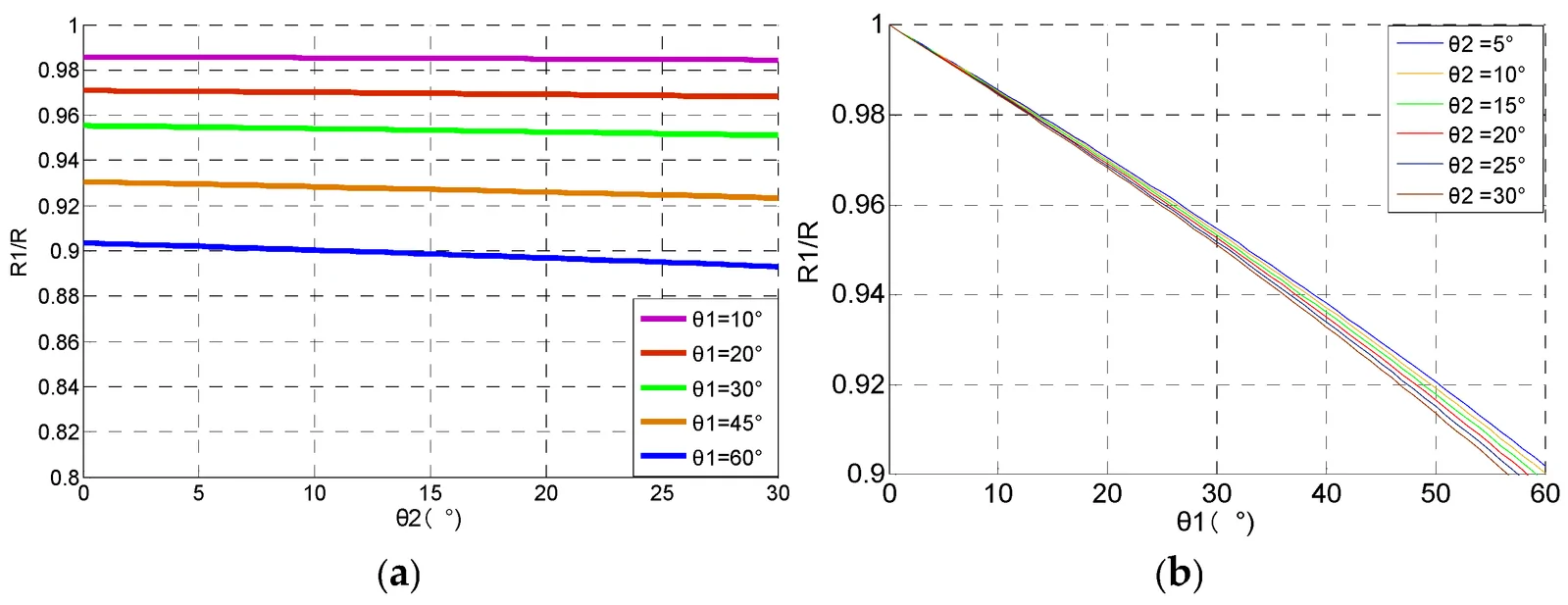
Relationship between the minimum detection range in sector 1~2N and *θ*_1_ and *θ*_2_, (**a**) with *θ*_2_ as the horizontal axis and (**b**) with *θ*_1_ as the horizontal axis.

**Figure 5:**
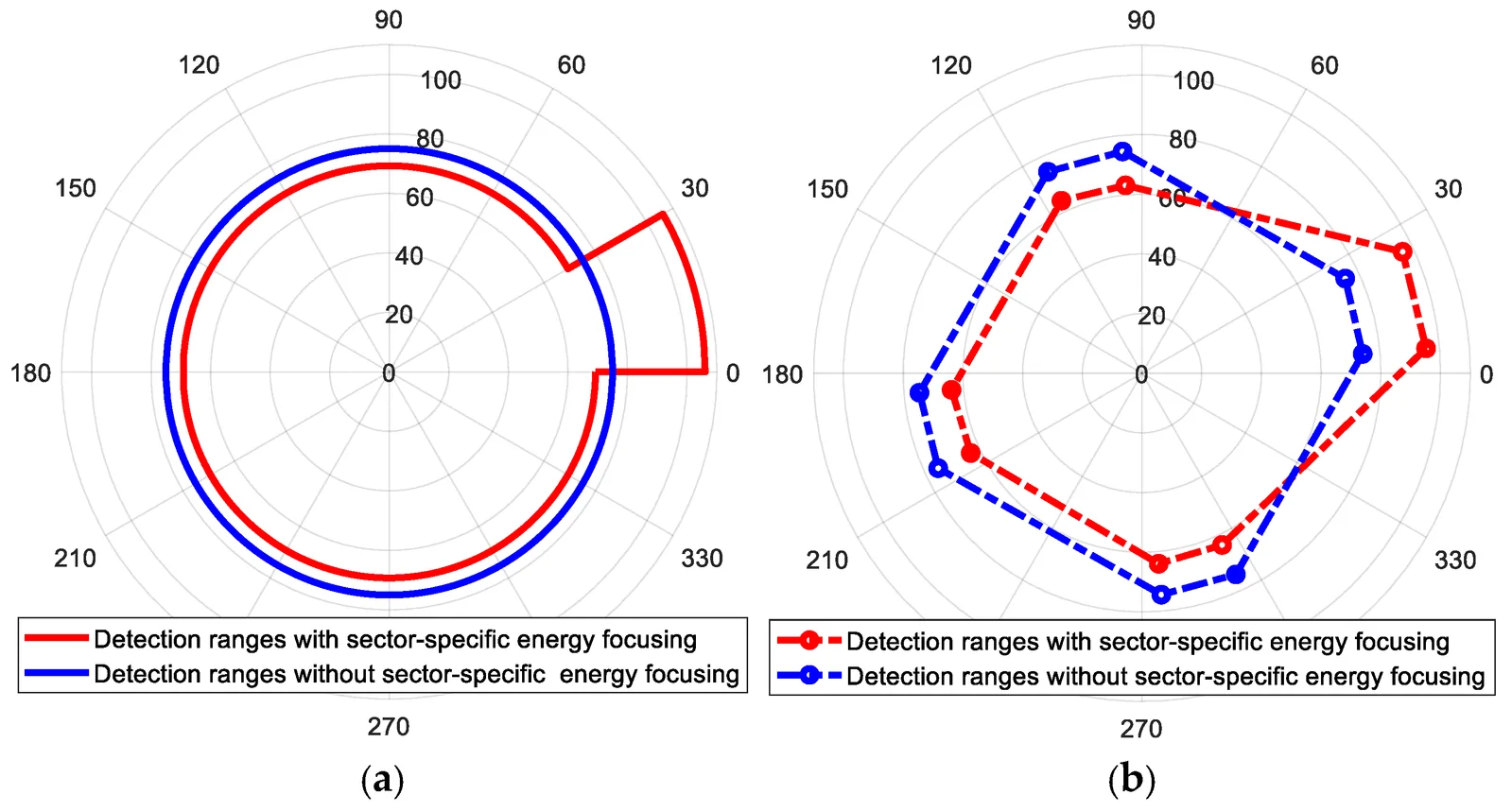
Theoretical and measured detection ranges in each sector for different modes. (**a**) Theoretical results; (**b**) measured results.

**Table 1:** Record of Experimental Results.

	Azimuth	5°	25°	95°	115°	185°	205°	275°	295°
Detection Range (km)									
Theoretical results without sector-specific energy focusing		75	75	75	75	75	75	75	75
Measured results without sector-specific energy focusing		74.2	75.1	74.6	74.5	74.8	75.3	74.5	74.4
Theoretical results with sector-specific energy focusing		106.1	106.1	69.3	69.3	69.3	69.3	69.3	69.3
Measured results with sector-specific energy focusing		95.5	96.3	63.2	63.8	64.0	63.3	64.1	63.5

## Data Availability

Data from this project will be made available upon request to the corresponding author.

## Appendix A

**Table A1:** Symbol Table.

Symbol	Definition
θ	The azimuthal beamwidth of the radar antenna
*t*	The dwell time per beam position
*R*	The detection range in each azimuthal direction
*θ*	The azimuthal span of sector 1 through N − 1 and sector N + 1 through 2N without energy focusing
*θ*_2_	The azimuthal span of sector 0 with energy focusing
*θ*_N_	The span of sector N
*θ*_s_	The maximum scanning angle of the antenna
*R*_1_	The detection range for sectors 1 through 2N without energy focusing
*R*_2_	The detection range for sector 0 with energy focusing
*t*_1_	The dwell (observation) time per beam position for sectors 1 through 2N
*t*_2_	The dwell (observation) time per beam position in sector 0
*T_n_*	The required duration for beam back-scanning from sector *n* to sector *n* − 1

## Appendix B

**Table A2:** Radar Parameter Table.

Parameter	Value	Unit
Operating frequency	1.3	GHz
Waveform	Linear frequency modulation (LFM)	—
Pulse repetition frequency (PRF)	0.3	kHz
Pulse width	100	μs
Bandwidth	5	MHz
Peak transmitted power	30	kW
Antenna gain	35	dBi
Polarization	Linear (horizontal)	—
Receiver noise figure	3.5	dB
Receiver bandwidth	6	MHz
Integration method	FFT-based Doppler filter bank	—
Detection threshold (CFAR)	P_fa = 10^−6^	—
